# Itaconate potentiates hepatic gluconeogenesis through NRF2 induction

**DOI:** 10.1371/journal.pone.0322946

**Published:** 2025-05-05

**Authors:** Marwa O. El-Derany, Sadeesh K. Ramakrishnan, Yingjie Li, Kathryn Buscher, Christina A. Jarad, Megan L. Schaller, Marc Cantwell, Thomas M. Vigil, Ryan A. Frieler, Peter Sajjakulnukit, Costas A. Lyssiotis, Richard M. Mortensen, Yatrik M. Shah

**Affiliations:** 1 Department of Molecular and Integrative Physiology, University of Michigan, Ann Arbor, Michigan, United States of America; 2 Department of Biochemistry, Faculty of Pharmacy, Ain Shams University, Cairo, Egypt; 3 University of Michigan Rogel Cancer Center, University of Michigan, Ann Arbor, Michigan, United States of America; 4 Department of Internal Medicine, University of Michigan, Ann Arbor, Michigan, United States of America; Hamadan University of Medical Sciences, IRAN, ISLAMIC REPUBLIC OF

## Abstract

The interplay between systemic metabolism and immune responses is increasingly recognized as a significant factor in the dysregulation of glucose homeostasis associated with diabetes and obesity. Immune metabolites play crucial roles in mediating this crosstalk, with itaconate emerging as an important immune metabolite involved in the inflammatory response of macrophages. Recent studies have highlighted the role of itaconate as a regulator of glucose metabolism, particularly in the context of obesity, although the underlying mechanisms remain poorly understood. In this study, we identified itaconate as one of the metabolites that significantly increase in the liver during fasting compared to fed conditions. Mechanistically, we found that itaconate enhances glucagon-induced liver gluconeogenesis independently of insulin signaling. Notably, itaconate upregulates the expression of gluconeogenic genes both under basal conditions and in the presence of palmitic acid. Furthermore, our data indicate that the effects of itaconate occur independently of CREB activation. Instead, we demonstrate that these potentiating effects are mediated through the induction of nuclear factor erythroid 2-related factor 2 (NRF2). Our findings demonstrate that itaconate has a glucagon-potentiating effects in the liver, suggesting that itaconate may play a significant role in the pathogenesis of metabolic-associated liver diseases.

## Introduction

Glucose is the primary energy source for cells, and its homeostasis must be tightly regulated to maintain metabolic health. The liver is central to this regulation, acting as a dynamic hub for glucose storage and production [1]. Two key hormones, insulin and glucagon, adjusts the metabolic activity in the liver to changes in nutrient availability. In the fed state, insulin is secreted by the pancreas, signaling the liver to store excess glucose as glycogen and to suppress gluconeogenesis. Insulin also promotes the conversion of glucose into fatty acids, which are exported to peripheral tissues for storage or energy use [2]. In contrast, during fasting, glucagon levels rise, signaling the liver to degrade glycogen and induce gluconeogenesis. The balance between these hormones allows adaptation to metabolic demands during feeding and fasting cycles. Disruptions in insulin and glucagon signaling can impair glucose regulation, leading to metabolic disorders such as diabetes, obesity and metabolic dysfunction-associated steatotic liver disease [3–5].

Metabolic diseases often involve complex immune interactions. Exploring small molecules that affect both immune and non-immune cells holds promise for new therapeutic interventions [6]. One such metabolite, itaconate, has gained significant attention for its role in metabolic reprogramming during immune responses [7]. Itaconate is produced from the tricarboxylic acid (TCA) cycle intermediate cis-aconitate by the enzyme immunoresponsive gene 1 (IRG1; also known as aconitate decarboxylase 1 (Acod1)) [8,9]. IRG1 is highly expressed in macrophages and is integral in regulating macrophage activation, polarization, and function, linking cellular metabolism, oxidative stress, and immune responses [7]. Recent studies highlight the role of IRG1 beyond immune regulation. IRG1 knockout mice displayed impaired glucose metabolism, with significant increases in blood glucose levels after 12 weeks on a high-fat diet (HFD) [10,11]. This metabolic dysfunction was accompanied by an increase in inflammatory gene expression and a decrease in genes related to adipogenesis and fatty acid metabolism [11]. In fact, the potential roles of IRG1 in the development non-alcoholic fatty liver diseases (NAFLD) remain unclear. While a recent study showed that itaconic acid is upregulated in human and mouse NAFLD models [12], others showed that IRG1 was significantly down-regulated in obesity-associated fatty liver [13]. These findings indicate that IRG1 not only influences immune responses but also plays a critical role in maintaining glucose homeostasis [11].

Itaconate interferes with TCA cycle intermediates, affecting cellular metabolism. Recent studies show that itaconate directly regulates fatty acid β-oxidation and the production of mitochondrial ROS, helping macrophages adapt to different functional states [12]. As an anti-inflammatory metabolite, itaconate and its cell permeable derivatives, such as dimethyl-itaconate and 4-octyl-itaconate, activate nuclear factor erythroid 2-related factor 2 (NRF2) in macrophages [7,14]. In addition to its cytoprotective role in oxidative stress, NRF2 suppresses the expression of pro-inflammatory genes such as IL-6 and IL-1β [15]. The antioxidant properties of itaconate are further underscored by its ability to induce glutathione production via NRF2 activation, which inhibits the accumulation of ROS. Through the induction of NRF2, itaconate emerges as a crucial regulator of both inflammation and oxidative stress [16].

Despite its prominent role in immune metabolism, the mechanistic impact of itaconate on glucose metabolism remains unexplored. Given its effects on lipid metabolism and glucose regulation in obesity, we aimed to investigate the mechanistic role of itaconate in glucose metabolism in primary hepatocytes under both fasting and fed conditions. In the present study, we found that itaconate predominantly increased in fasting conditions as compared to the fed state. Mechanistically, we found that itaconate potentiates glucagon effects in the presence and absence of palmitic acid. This potentiation effects was dependent on NRF2.

## Methods

### Mice and diets

Wild type (WT) C57BL/6J mice, *Nrf2*^F/F^ were either bred in-house or purchased from Jackson Laboratories. *Irg1*^fl/fl^ mice with loxP sites flanking exon 5 were used [11]. These animals were crossed with *Mrp8*-Cre, *LysM*-Cre and Alb-Cre from Jackson Laboratory. WT mice were fed with either normal chow or 60% high fat diet (40kcal mostly palm oil, 20 kcal% Fructose and 2% Cholesterol) (Research diets, New Brunswick, NJ) for four months. For fasting experiment, mice were fasted overnight, followed by refeeding for 2 hours. All mice were housed at the Unit for Laboratory Animal Management (ULAM) at the University of Michigan. All animal procedures were approved by the University of Michigan Institutional Animal Care and Utilization Committee (IACUC). All mice were fed *ad-libitum* and kept in a 12-hour dark/light cycle with chow replenished every week. Mice were euthanized by inhalation of carbon dioxide gas and then by cervical dislocation to confirm euthanasia. Animal studies were carried out in accordance with Association for Assessment and Accreditation of Laboratory Animal Care International guidelines. Moreover, they were approved by the University Committee on the Use and Care of Animals at the University of Michigan.

### Metabolomic analysis

Livers were collected and samples were normalized by mass of liver tissue. Metabolites were extracted by adding cold 80% methanol for 10 minutes then homogenized. Samples were centrifuged via high-speed centrifugation and the resulting supernatant was extracted. Lyophilization for the metabolite extracts were done using a SpeedVac concentrator. Lyophilized samples were resuspended in 50:50 methanol/water mixture for LC-MS analysis. Data was collected in both negative and positive ion modes. Agilent Masshunter Workstation Software LC/MS Data Acquisition for 6400 Series Triple Quadrupole MS with Version B.08.02 is used for calibration, optimization and data acquisition. A Waters Acquity UPLC HSS T3 1.8 mm VanGuard Pre-Column 2.1 x 5 mm column and a Waters UPLC BEH TSS C18 column (2.1 x 100mm, 1.7mm) was used with mobile phase A). It consists of 0.1% formic acid in water; mobile phase (B) consists of 0.1% formic acid in acetonitrile. For both ion modes QQ data were preprocessed with Agilent MassHunter Workstation Quantitative Analysis Software (B0700). Chromatogram peaks for metabolites were manually inspected and metabolite abundance level was median normalized across the sample population. Statistical significance was determined by a one-way ANOVA with a significance of 0.05. Metaboanalyst was used for pathway enrichment analysis.

### Primary hepatocytes isolation and treatment

Primary hepatocytes were isolated from the mice on normal chow diet as previously described [17]. Briefly, livers were perfused with 10 ml of HBSS containing 0.5 mM EGTA, followed by perfusion with HBSS containing collagenase type II (Worthington, Lakewood, NJ). Viable cells were plated in DMEM media with 10% FBS and 1% antibiotic-antimycotic. For dimethyl itaconate (DMI) treatment, primary hepatocytes were FBS-starved overnight and treated with DMI (250 μM) for three hours then cells were treated with either glucagon (50 nM) or insulin (10 nM) for 6-hours (RNA analysis) or 10-minutes (protein analysis). Then cells were washed twice with PBS and lysed with either Trizol for RNA extraction or RIPA buffer for protein extraction. Experiments were done in triplicates and repeated at least three times.

### Measurements of glucose levels

Primary hepatocytes were isolated as previously described and cultured overnight in Williams E media with 10% FBS. Cells were then washed by PBS three times and incubated with Krebs-Ringer Bicarbonate Buffer (KRB) (NaCl 119 mM, KCl 5 mM, KH 2 PO 4 2.6 mM, CaCl 2 2 mM, NaHCO 3 24.6 mM, MgSO 4 2.6 mM and HEPES 10 mM, pH 7.4) for 2 hours. Cells were then treated with glucagon 50nM and or DMI 250 mM and compared to the untreated cells for 4 hours. Amount of glucose released in the KRB buffer is measured using glucose kit (Stanbio Laboratories, Boerne, TX). Glucose production was normalized by protein content released from lysed cells and the values are expressed as μg/mg of protein/hour as previously described [18].

### Bone Marrow-derived macrophage (BMDM) isolation and treatment

Isolation was done as previously described [19]. Briefly, the femur bones were dissected from wild type mice, muscles were removed, end of bones were cut and the bone marrow was extracted by flushing with 10 mL RPMI media containing 10% FBS, 1% antibiotic-antimycotic and 20 ng/mL GM-CSF using a 25-gauge needle in a 10 cm petri dish. The suspension was then centrifuged at 250g for 5 minutes to pellet the cells. The pelleted cells were resuspended in 10 mL of sterile water to lyse all the erythroid progenitor cells, then 1 mL of 10X PBS was added. Cells were counted and plated at a cell density of 2x10^6^ cells/10 mL in a petri dish. The cells were plated passaged and on day 10 and were treated with either glucagon (50 nM) or insulin (10 nM) for 6-hours for RNA analysis. Then cells were washed twice with PBS and lysed with Trizol for RNA extraction or RIPA buffer. Experiments were done in triplicates and repeated at least three times.

### Gene expression analysis

RNA was extracted using Trizol/chloroform method according to the manufacturer’s protocol. cDNA was converted from 1 μg of the purified RNA using the High-Capacity cDNA Reverse Transcription Kit and qPCR was performed as previously described. Each reaction was performed in triplicate and expression levels of all genes were normalized to those of *Gapdh* using the ΔΔCt method.

**Table:** 

G6Pase F	GTGTCCAGGACCCACCAATA
G6Pase R	ACTGTGGGCATCAATCTCCT
PEPCK F	CTGGATGAAGTTTGATGCCC
PEPCK R	TGTCTTCACTGAGGTGCCAG
NRF2 F	GGACATGGAGCAAGTTTGGC
NRF2 R	CCAGCGAGGAGATCGATGAG
IRG1 F	GCGAACGCTGCCACTCA
IRG1 R	ATCCCAGGCTTGGAAGGTC
GAPDH F	TTGATGGCAACAATCTCCAC
GAPDH R	CGTCCCGTAGACAAAATGGT

### Chromatin bound protein fraction

Cells were harvested, spun down and pellets were resuspended in buffer A (10mM HEPES, pH7.9, 10mM KCL, 1.5mM MgCl2, 0.34M sucrose, 10% glycerol, ImM DTT and protease inhibitor cocktail) + 0.1% Triton X-100 and incubated on ice for 12 min. The mixture was centrifuged at 1300xg for 5min at 4C and pellets and resuspended with Buffer B (3mMEDTA, 0.2mM EGTA, 1mM DTT and protease inhibitor cocktail) and incubated for 30 min on ice. The mixture was then centrifuged at 1700x g for 5 min at 4C and pellets were washed with Buffer B + 150mM NaCl. Pellets were then resuspended in RIPA + protease inhibitor cocktail and incubated on ice then sonicated at 20% amplitude for 10 sec twice. Chromatin fraction was then clarified by high speed centrifugation 10,000x g for 5 min at 4C and the supernatant were collected as the final chromatin fraction [20].

### Immunoblotting

Lysates were prepared by homogenizing the cells in RIPA buffer (150 mM NaCl; 1% NP-40; 0.5% C_24_H_39_O_4_Na; 0.1% SDS; 50 mM Tris–HCl, pH 8.0). Then the lysates were sonicated and centrifugation (20,000x *g*) for 10 min at 4C. Protein quantification was determined by BCA assay (Pierce) kit. Lysates were e resolved by SDS-PAGE and transferred using standard procedures.

Primary antibodies pAKT-S473, AKT, pS6 Ser 240/244, S6, pCREB Ser 133, CREB and GCLM (these antibodies were from Cell Signaling), β-Actin and Vinculin (Proteintech) and Histone H1, G6pase and PEPCK (Santa Cruz Biotechnology). HRP-conjugated secondary antibodies were purchased from Cell signaling.

### Statistical analysis

Results were expressed by mean ± SEM. For analysis of more than two groups, statistical significance was estimated by One-way ANOVA followed by Tukey’s Post Test. T test was applied to find statistical significance for two group comparisons. P < 0.05 was considered to be statistically significant. The graphpad prism 19 was used for data analysis

## Results

### Liver itaconate increases during fasting

**Fig 1:**
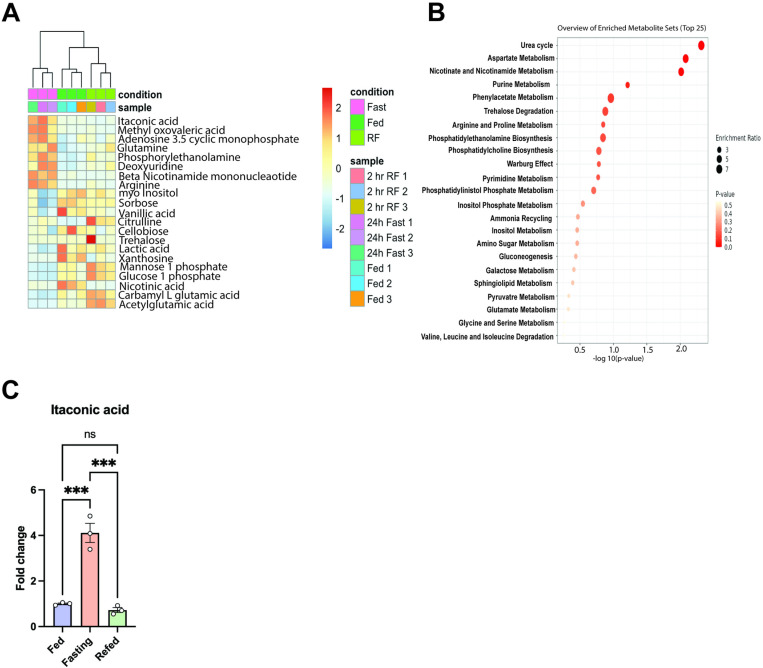
Liver Itaconate increases following fasting. **A)** Heat map, **B)** Pathways set of enriched metabolite set during fasting mouse models (n = 3), and **C)** Itaconate levels in fasting, fed and refed conditions. Fed mice were on regular chow diet, fasting mice were fasted overnight, refed mice were fasted overnight followed by refeeding for 2 hours. Each bar represents the mean value ± S.E.M. *p < 0.05, **p < 0.01, ***p < 0.001 as analyzed by One-way ANOVA followed by Tukey’s Post Test.

Fasting mice underwent 12 hours of food deprivation, receiving only water, while the fed group was allowed access to standard food and water. Following fasting, the mice were euthanized, and liver metabolomics was performed (Fig 1A). Metabolite set enrichment analysis revealed an increase in the urea cycle, aspartate and phenylacetate metabolism in the fasting state as compared to the fed state. Moreover, there was a depletion of valine, leucine, isoleucine, glycine, and serine metabolism (Fig 1B). Itaconate was the top metabolite that significantly increased during fasting as compared to the fed mice. Furthermore, measurements of tissue itaconate levels confirmed an increase in the livers of fasted mice relative to their fed counterparts (Fig 1C). While modulation of the TCA cycle occurs during fasting, the increase in itaconate has not been reported. This data suggests that itaconate is involved in glucose homeostasis during fasting. This aligns with recent studies that shed lights on the possible glucose regulatory role of itaconate [11,12].

### IRG1 expression increases in fasting and high fat diet (HFD) fed mice

**Fig 2:**
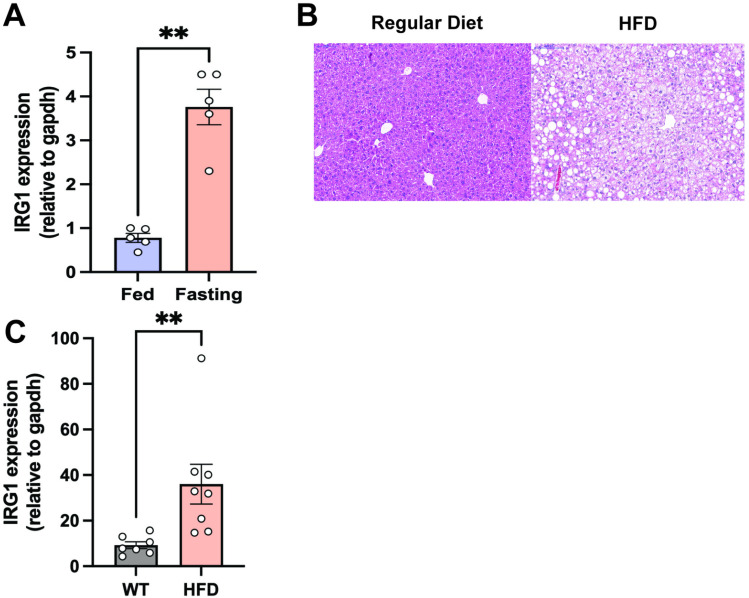
Liver IRG1 increases in fasting and following high fat diet (HFD). IRG1 gene expression in whole livers of **A)** fasting and fed mice (n = 5) and **C)** following HFD for 4 months (n = 7). **B)** Hematoxylin and eosin stained sections of liver in mice on **c**ontrol diet or HFD. Each bar represents the mean value ± S.E.M. **p < 0.01 as analyzed by two-tailed *t t*est.

Gene expression analysis in fasting livers showed significant increase in IRG1 gene expression as compared to fed mice (Fig 2A). The gene expression data aligns with our metabolite quantification analysis. Previous studies have demonstrated that IRG1-deficient mice exhibit increased hepatic lipid accumulation, while treatment of primary hepatocytes with itaconate, the metabolite produced by IRG1, reduces lipid deposition and enhances fatty acid oxidation. Despite these findings, IRG1 expression is upregulated in hepatic macrophages in response to a HFD [12], and similarly, whole-liver analyses have revealed increased IRG1 expression in HFD-fed mice [11]. Given this prior work, we examined IRG1 expression in a metabolic dysfunction-associated steatotic liver disease (MASLD) mouse model induced by HFD and compared it to normal chow-fed controls. After four months on their respective diets, HFD-fed mice exhibited macroscopic and histological evidence of steatosis (Fig 2B). Consistent with prior reports, IRG1 expression was significantly elevated in the livers of HFD-fed mice relative to controls (Fig 2C). This induction suggests an adaptive response aimed at mitigating lipid accumulation, aligning with previous studies showing IRG1 upregulation in both human and murine MASLD models [11,12].

### Itaconate is primarily secreted from macrophages

**Fig 3:**
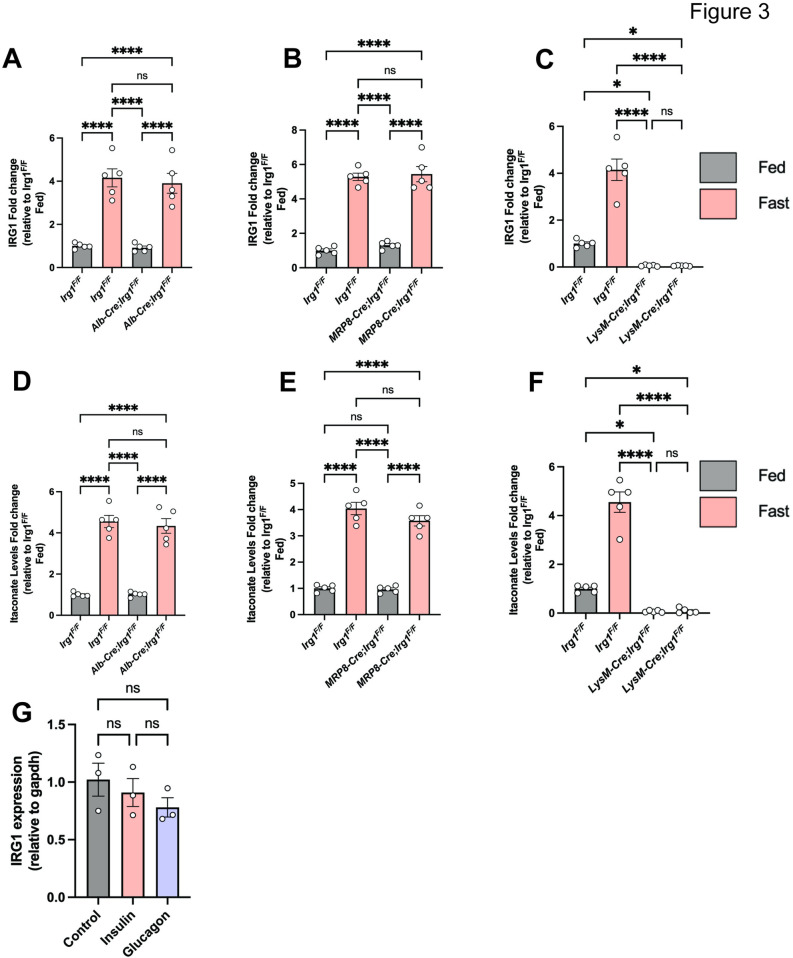
Itaconate during fasting is primarily produced from hepatic macrophages. Liver IRG1 expression in fed and fasted mice in IRG1 **A)** hepatocyte, **B)** neutrophil, and **C)** macrophage knockout mice (n = 5). Liver itaconate levels in fed and fasted mice in IRG1 **D)** hepatocyte, **E)** neutrophil, and **F)** macrophage knockout mice (n = 5). **G)** Mouse primary macrophages were serum starved overnight then stimulated with glucagon (50 nM) for 6 hours or insulin (10nmol) then *IRG1* gene expression was assessed. Each bar represents the mean value ± S.E.M. *p < 0.05, **p < 0.01, ***p < 0.001 as analyzed by One-way ANOVA followed by Tukey’s Post Test.

Macrophages are the primary source of itaconate production [7,21]. Previous studies have shown that in macrophages itaconate regulates the inflammatory response as well as β-oxidation of lipids which promoted mitochondrial reactive oxygen species (ROS) production and oxidative phosphorylation [12,22,23]. However, these effects have predominantly been observed under conditions of inflammatory stimuli that activate macrophages, such as lipopolysaccharide treatment [24]. To determine the cell type that increases itaconate under fasting, we generated conditional knockout mice for IRG1, by crossing *Irg1*^F/F^ mice to LysM-Cre (macrophage), Mrp8-Cre (neutrophils), or Alb-Cre (hepatocytes). *Irg1* expression was induced in the liver following fasting in all littermate control mice (Fig 3A–3C). Interestingly, disruption of *Irg1* in hepatocytes or neutrophils did not decrease basal or fasting-induced *Irg1* expression in the liver (Fig 3A and 3B). Disruption of *Irg1* in macrophages decreased basal Irg1 expression in the liver, and decreased *Irg1* expression in fasting liver as well (Fig 3C). Consistent with the *Irg1* expression data, macrophages were identified as the primary source of both basal and fasting-induced itaconate. (Fig 3D–3F). These findings align with previous reports that macrophages are the major source of itaconate [25,26]. Additionally, we wanted to assess the mechanism by which *Irg1* gene expression is induced by fasting in macrophages. We isolated bone marrow-derived macrophages and treated them with insulin and glucagon, but no change in *Irg1* gene expression was observed (Fig 3G). Our data clearly shows that fasting induces the *Irg1*-itaconate axis in macrophages, but this response is not directly mediated by insulin or glucagon.

### Itaconate does not alter insulin action

**Fig 4:**
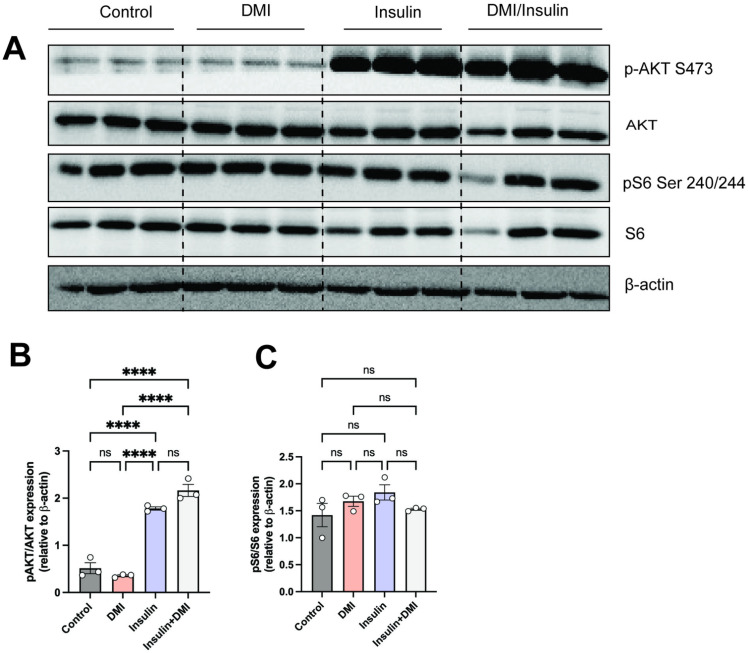
Itaconate does not alter liver insulin signaling. Mouse primary hepatocytes were serum starved followed by pretreatment of DMI (250uM) for 3 hours and then cells were stimulated with insulin (10nM) for 10 min. **A)** Westerns and **B-C)** quantification of phospho AKT (p-AKT S473)/ total AKT or phospho-S6 (p-S6 Ser 240/244)/ total S6. β-actin was used as a loading control. The experiments with primary hepatocytes were done in triplicates (n = 3) and repeated at least three times.

Previous studies have demonstrated that itaconate improves glycemic deterioration, abrogates the increased systemic insulin level, and improves glucose metabolism in diabetic mice and non-obese diabetic mice [27]. Moreover, it was shown that itaconate intervention significantly improves beta cell function, consistent with improved systemic glucose metabolism and less immune cell infiltration as compared to the diabetic mice [27]. *Irg1* knockout mice were shown to have significant increase in insulin tolerance and glucose tolerance after 12 weeks on HFD [11]. We wanted to study the potential effect of itaconate on primary hepatocytes during fasting and fed conditions, therefore we utilized dimethyl itaconate (DMI), which is a known cell permeable derivative of itaconate [14]. Primary hepatocytes isolated and serum starved overnight. Insulin mediated activation of downstream pathways was not altered by pretreatment with DMI (Fig 4), suggesting that itaconate has no effect on hepatic insulin signaling.

### Itaconate potentiates glucagon effect

Based on our metabolomics analysis which showed that itaconate was significantly increased under fasting conditions we then assessed the effect of itaconate on hepatic glucagon response.

**Fig 5:**
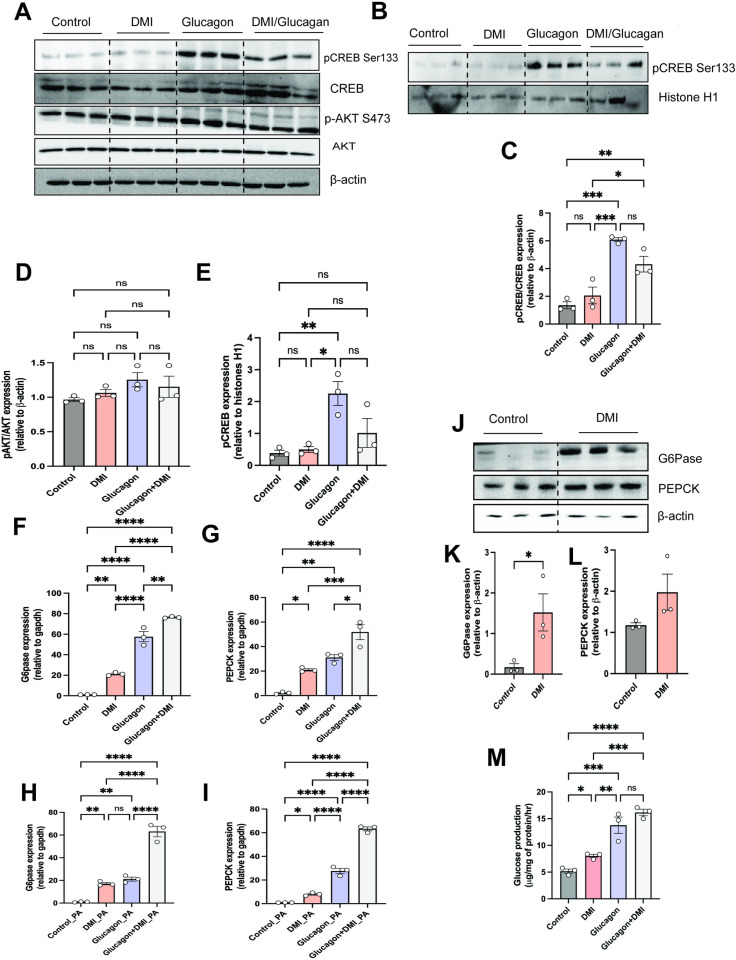
Itaconate potentiates liver glucagon-mediated gene expression. Mouse primary hepatocytes were serum starved followed by addition of DMI (250μM) for 3 hours and then stimulated with glucagon (50 nM) for 10 min, **A)** whole cell lysates were assessed by western blot analysis, **B)** Chromatin bound fractions were assessed by Western blot analysis, **C& D)** quantification of phospo-CREB (p-CREB Ser 133)/ total CREB or phospho AKT (p-AKT S473)/ total AKT. **E)** quantification of chtomatin phosphor-CREB(p-CREB Ser 133)/histone H1. **F and G)** Mouse primary hepatocytes were serum starved followed by addition of DMI (250μM) for 3 hours and then stimulated with glucagon (50 nM) for 6 hours or **H and I)** prior to DMI treatment the cells were treated with palmitic acid (250μM). **F, and H)** *G6pase* and **G and I)** *Pepck* gene expression was assessed (n = 3). **J)** Westerns for G6Pase and Pepck and **K-L)** quantification of G6Pase or Pepck. β-actin was used as a loading control. **M)** Glucose production in primary hepatocytes pretreated for 1 hr with or without glucagon (50 nM) or DMI (250mM).The experiments with primary hepatocytes were done in triplicate and repeated at least three times The experiments with primary hepatocytes were done in triplicate and repeated at least three times. Each bar represents the mean value ± S.E.M. *p < 0.05, **p < 0.01, ***p < 0.001 as analyzed by One-way ANOVA followed by Tukey’s Post Test.

Glucagon induces gluconeogenic genes through the activation of the transcription factor CREB. CREB is activated by phosphorylation at serine 133, leading to its binding to response elements on the promoters of gluconeogenic genes [28]. However, our results showed no significant change in AKT phosphorylation (Fig 5A,5D). Our results showed significant increase in pCREB expression following glucagon treatment with no change with DMI and glucagon combination (Fig 5A,5C). In addition to phosphorylation, CREB is regulated by acetylation and other posttranslational modifications [29]. To directly assess CREB activity we isolated the chromatin bound fraction of CREB following glucagon and DMI treatment and then performed Western analysis. Our results showed no significant change in CREB bound chromatin between glucagon and glucagon and DMI group (Fig 5B and 5E).

Glucose 6 phosphatase (G6Pase) and phosphoenol pyruvate carboxykinase (Pepck) are the key gluconeogenic genes induced by glucagon in fasting conditions [30]. We assessed their gene expression in mouse primary hepatocytes treated with DMI and glucagon. DMI increased the gene expression of *G6pase* and *Pepck*, and this was further potentiated following of glucagon treatment (Fig 5F and 5G). Multiple studies have highlighted the role of itaconate in lipid metabolisms [10,11,31]. Therefore, we assessed the potentiation effect of itaconate on glucagon response under high palmitoleic acid to mimic HFD fed mice. Our results found a significant increase in the expression of *G6pase* and *Pepck* genes with DMI and glucagon combination as compared to glucagon alone (Fig 5H and 5I). Moreover, we determined the protein level of G6pase in DMI treated cells and as compared to the untreated were significantly increased (Fig 5J and 5K). An increase in PEPCK protein levels was also observed in DMI treated condition, although did not reach significance (Fig 5J and 5L). Additionally, we assessed the glucose production in primary hepatocytes treated with glucagon and/ or DMI and found a significant increase in glucose with DMI and with glucagon (Fig 5M).

### Itaconate potentiates glucagon effect through NRF2

**Fig 6:**
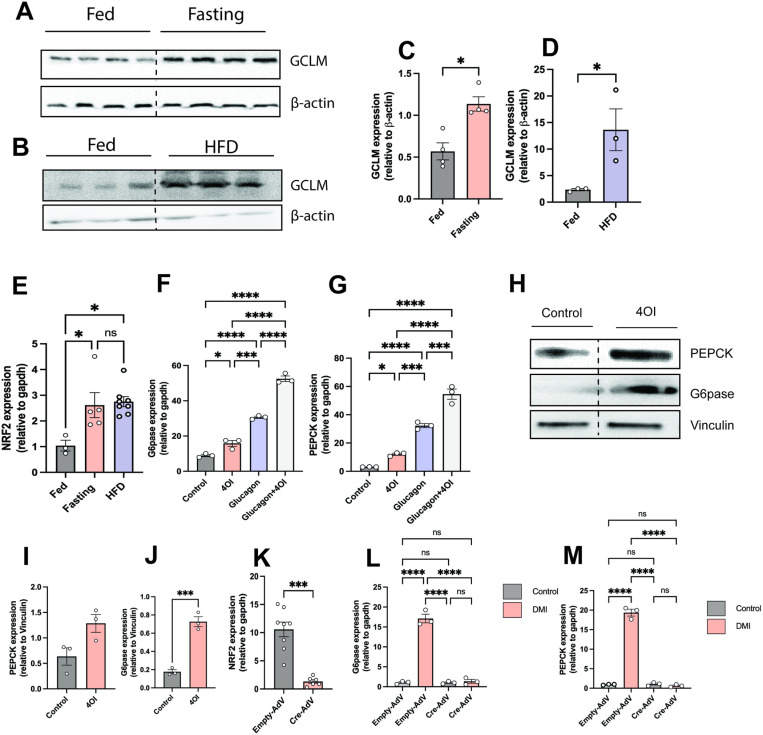
Itaconate potentiates liver glucagon signaling through NRF2. **A and B)** Western blot and **C)** quantification of GCLM/β-actin in livers of fed and fasting mice (n = 4), **D)** quantification of GCLM/β-actin in livers of fed and HFD mice (n = 3) **E)** NRF2**, F and G)** Mouse primary hepatocytes were serum starved followed by addition of 4OI (250μM) for 3 hours and then stimulated with glucagon (50 nM) for 6 hours**. F)** *G6pase* and **G)** *Pepck* gene expression was assessed (n = 3). **H)** Westerns for Pepck and G6Pase and **I-J)** quantification of Pepck and G6Pase. Vinculin was used as a loading control. **K)** NRF2 gene expression in *Nrf2*^F/F^ infected with empty adeno associated virus (AAV), **L)** G6pase and **M)** Pepck gene expression in primary hepatocytes isolated from *Nrf2*^F/F^ infected with empty adeno associated virus (AAV) or AVV expressing Cre recombinase (n = 6), followed by serum starvation and addition of DMI (250μM) for 6 hours (n = 3). The experiments with primary hepatocytes were done in triplicate and repeated at least three times. Each bar represents the mean value ± S.E.M. *p < 0.05, **p < 0.01, ***p < 0.001 as analyzed by One-way ANOVA followed by Tukey’s Post Test.

Previous work demonstrated that itaconate activates NRF2 [32]. Interestingly, a recent report showed that NRF2 is involved in glucose uptake and glucose metabolism [33]. Our results found increased in glutamate-cysteine ligase modifier subunit (GCLM), a direct target of the Nrf2 transcription factor [34], in fasting and HFD livers as compared to regular chow fed mice (Fig 6A–6D). We also found increase in NRF2 gene expression in fasting and HFD livers as compared to regular chow fed diet mice (Fig 6E). DMI has multiple targets, we wanted to check another cell permeable itaconate derivative, 4 Octyl itaconate (4OI), that is known to activate NRF2 [32]. So we assessed the effect of 4OI on gluconeogenesis genes and we found increased expression of G6Pase and *Pepck* gene following treatment with 4OI (Fig 6F and 6G). An increase in PEPCK protein levels was also observed in 4OI treated condition, although did not reach significance (Fig 6H and 6I). Moreover, we determined the protein level of G6pase in 4OI treated cells and as compared to the untreated were significantly increased (Fig 6H and 6J).

Thus, we aimed to investigate the association between the glucagon potentiation effects of itaconate and NRF2 induction. Primary hepatocytes were isolated from *Nrf2*^F/F^ mice and an adenovirus expressing Cre was used to disrupt *Nrf2*. We confirmed a decreased expression of *Nrf2* following Cre infection compared to a control virus (Fig 6K). Moreover, we demonstrated that *G6pase* and *Pepck* induction by DMI was dependent on NRF2 (Fig 6L and 6M). This finding sheds light on how itaconate enhances the effects of glucagon by inducing NRF2.

## Discussion

Emerging evidence indicates that immune metabolites may play a role in nearly all metabolic diseases, and understanding how the inflammatory and metabolic pathways interact is critical for identifying potential therapeutic interventions [35]. Recently, it was demonstrated that itaconate plays a critical role in regulating glucose homeostasis and obesity under HFD [11]. These findings established the first connection between itaconate and glucose dysregulation in obesity. More recently, itaconate treatment was shown to regulate systemic glucose homeostasis, preventing glucose intolerance and insulin resistance in HFD-fed mice [11]. In this study, oral glucose tolerance tests and insulin tolerance tests were performed after 30 days of itaconate administration, concurrent with HFD feeding. Itaconate-treated mice showed significantly reduced fasting glucose, lower serum insulin levels, improved glucose tolerance, and decreased insulin resistance compared to vehicle controls [36]. Collectively, these results highlight the pivotal role of itaconate in regulating blood glucose homeostasis. Our metabolic screening of fed and fasted mice revealed that itaconate is significantly induced during fasting compared to fed conditions in the liver. We observed a marked increase in IRG1 expression in the livers of fasting mice compared to those in the fed state. Similarly, hepatic IRG1 expression was significantly elevated in mice fed a HFD relative to those on a normal chow diet. These findings are consistent with recent studies highlighting a role for IRG1 in glucose metabolism during obesity, which reported elevated hepatic IRG1 levels in obese mice [11]. Building on this, we explored the direct effects of itaconate on glucose metabolism in primary hepatocytes and elucidated the molecular mechanisms through which itaconate exerts its regulation on glucose homeostasis. We found that itaconate may play a regulatory role in fasting, potentially through augmentation of glucagon mediated effects on gluconeogenesis independent of its effect on insulin. Additionally, we found increased GLCM protein levels in fasting livers as compared to normal fed mice. Being a direct target of NRF2, increase expression of GCLM protein means that Nrf2 is activated in fasting livers [37].

As an immune metabolite, our findings align with previous reports indicating that itaconate is primarily produced by macrophages, where it exerts anti-inflammatory effects [38]. In our study, livers of macrophage-specific IRG1 knockout mice showed no changes in itaconate levels during fasting, while hepatocyte, endothelial, and neutrophil IRG1 knockouts exhibited increased itaconate levels in fasting compared to fed mice. Itaconate was significantly upregulated by macrophages during the inflammatory response, which led to a potent anti-inflammatory function, linking cellular metabolism to innate immunity [25]. However, the mechanism that increased the IRG1-itaconate axis during the fed/fasting cycle has not yet been determined. Although there are systemic changes in insulin and glucagon levels during fed/fasting cycle, the direct addition of these hormones did not alter macrophage IRG1 levels, suggesting a novel activation mechanism. This connection between fasting, inflammation, and itaconate opens new avenues for research into the immune-metabolic interactions during fasting.

We investigated whether itaconate modulates the response to insulin and glucagon, the key hormones regulating metabolic transitions between fed and fasted states. Previous research has shown that itaconate improves palmitate-induced insulin resistance [11]. However, our findings indicate that itaconate does not directly influence insulin signaling in the liver. This suggests that itaconate’s effects on insulin resistance may instead arise due to cell-specific response of pancreatic islets to itaconate through indirect mechanisms, such as enhancing liver glucagon signaling to inhibit insulin resistance, or potentially mediated through the regulation of inflammatory pathways; this is consistent with earlier reports that highlight itaconate’s anti-inflammatory properties across various disease models [39–41]. This aligns with a prior report which showed that DMI attenuates palmitate-induced insulin resistance in skeletal muscle cells through suppressing inflammation [40].

Our study demonstrated that itaconate enhanced glucagon signaling by upregulating the expression of key rate-limiting enzymes involved in gluconeogenesis. Notably, this potentiation of glucagon does not appear to involve the classical CREB pathway, instead, our data suggest that itaconate potentiates glucagon signaling through NRF2. Disruption of NRF2 abolishes itaconate-induced upregulation of the rate-limiting gluconeogenic enzymes G6Pase and Pepck. This is supported by recent studies that showed NRF2 promotes glucose uptake and metabolism in neuronal cells and astrocytes [33]. Additionally, increased NRF2 expression and activation of its downstream targets have been observed in diabetic patients with elevated glucose levels, underscoring the role of NRF2 in glucose metabolism [42]. Additionally, our results highlighted increased expression of GCLM protein in fasting livers as compared to regular fed mice which highlights NRF2 activation in fasting. In alignment, previous studies pointed toward the possible regulation of NRF2 signaling pathways by the nutrient status [43]. Whereas, it was found that fasting induced the activation of NRF2 and its downstream antioxidant pathway [43]. Moreover, the genetic activation of Nrf2 was found to protect against fasting-induced oxidative stress [19]. Others also agreed on increased expression of NRF2 in fasting in overweight and obese subjects [44]. However, further studies are required to elucidate the precise molecular mechanisms by which NRF2 influences gluconeogenesis and its interaction with itaconate.

Our results revealed that itaconate significantly enhances glucagon-induced gluconeogenesis through NRF2 induction. This positions itaconate as a potential therapeutic target in diabetes and obesity-related disorders, highlighting its critical role at the intersection of inflammation and metabolic regulation. Targeting this pathway could offer therapeutic benefits for patients with these metabolic conditions.
